# Infrared receptors in pyrophilous (“fire loving”) insects as model for new un-cooled infrared sensors

**DOI:** 10.3762/bjnano.2.22

**Published:** 2011-03-30

**Authors:** David Klocke, Anke Schmitz, Helmut Soltner, Herbert Bousack, Helmut Schmitz

**Affiliations:** 1 Institute of Zoology, University of Bonn, Poppelsdorfer Schloss, 53115 Bonn, Germany; 2 Forschungszentrum Jülich GmbH, Zentralabteilung Technologie, 52425 Jülich, Germany; 3 Forschungszentrum Jülich GmbH, Peter Grünberg Institut, 52425 Jülich, Germany

**Keywords:** fire detection, forest fire, Golay cell, infrared sensor, pyrophilous insects

## Abstract

Beetles of the genus *Melanophila* and certain flat bugs of the genus *Aradus* actually approach forest fires. For the detection of fires and of hot surfaces the pyrophilous species of both genera have developed infrared (IR) receptors, which have developed from common hair mechanoreceptors. Thus, this type of insect IR receptor has been termed photomechanic and shows the following two special features: (i) The formation of a complex cuticular sphere consisting of an outer exocuticular shell as well as of a cavernous microfluidic core and (ii) the enclosure of the dendritic tip of the mechanosensitive neuron inside the core in a liquid-filled chamber. Most probably a photomechanic IR sensillum acts as a microfluidic converter of infrared radiation which leads to an increase in internal pressure inside the sphere, which is measured by a mechanosensitive neuron.

A simple model for this biological IR sensor is a modified Golay sensor in which the gas has been replaced by a liquid. Here, the absorbed IR radiation results in a pressure increase of the liquid and the deflection of a thin membrane. For the evaluation of this model analytical formulas are presented, which permits the calculation of the pressure increase in the cavity, the deformation of the membrane and the time constant of an artificial leak to compensate ambient temperature changes. Some organic liquids with high thermal expansion coefficients may improve the deflection of the membrane compared to water.

## Introduction

Fire loving (pyrophilous) insects depend on forest fires for their reproduction. Such insects approach ongoing fires and invade the burnt area immediately after a fire. For the long-range navigation toward a fire as well as for the short-range orientation on a freshly burnt area these insects have special sensors for smoke and infrared (IR) radiation. Whereas the olfactory receptors for smoke are located on the antennae, the IR receptors are housed in extra-antennal sensory organs, which can be found on the thorax or on the abdomen. In the pyrophilous beetle *Melanophila acuminata* infrared receptors and their associated sensory neurons are derived from mechanoreceptors [1]. Unlike other mechanosensory neurons, IR sensitive neurons directly send their information to be processed centrally (e.g., by the brain) rather than locally in their respective ganglia of origin [2]. It is suggested that smoke-derived odours and IR information converge on descending brain neurons which, in turn, control and direct flight toward the forest fire.

Two genera of jewel beetles (family Buprestidae) can be classified as pyrophilous: About a dozen species of the genus *Melanophila,* which are distributed nearly all over the world except for Australia, and the ”fire-beetle” *Merimna atrata,* which is endemic to Australia [3–4]. Despite the fact that *Melanophila* and *Merimna* show almost the same behaviour and belong to the same family of jewel beetles, their IR receptors are very different from each other.

On a freshly burnt area, the males of both genera often stay on the stems of trees close to burning or glowing wood or hot ashes. As soon as they become aware of a conspecific female, they try to copulate vigorously. After mating, the females deposit their eggs under the bark of burnt trees. The main reason for the pyrophilous behaviour is that the wood-boring larvae of *Melanophila* and *Merimna* can only develop in the wood of burnt trees [3,5]. As a morphological speciality, both pyrophilous buprestid genera are equipped with antennal smoke receptors and thoracic or abdominal IR organs [6–9].

Another pyrophilous beetle can also be found in Australia, i.e., the “little ash beetle” *Acanthocnemus nigricans* (family Acanthocnemidae). This inconspicuous beetle is only 4 mm long and highly attracted by hot ashes: Little is known about its biology. Similarly, *Acanthocnemus* also depends on fires for its reproduction and is equipped with a pair of sophisticated prothoracic IR receptors [10–11]. Recently, IR receptors have also been discovered in a few pyrophilous members of the flat bug genus *Aradus* (Heteroptera, Aradidae) [12]. With respect to morphology and function, the IR receptors of *Aradus* bugs are very similar to those described for *Melanophila* beetles. Fire detection is obviously an important requirement for the survival of all of the pyrophilous insect species noted above. However, the outbreak of a forest fire is highly unpredictable. Therefore, pyrophilous beetles and bugs must be able to detect fires from distances as large as possible. Furthermore, when flying over a burnt area in search for a place to land, the small insects have to avoid “hot spots” with dangerous surface temperatures above about 60 °C.

*Melanophila* beetles and *Aradus* bugs are equipped with sensory structures that allow both the detection of hot fires at considerable distances as well as being able to locate moderately hot spots close by. These insects feature so-called photomechanic IR receptors, which might serve well as models for the technical design of un-cooled IR receptors. In the present paper we focus on the structure and function of the biological model as well as on the design and theoretical evaluation of a technical photomechanic IR sensor.

## Results

### The photomechanic IR receptors in pyrophilous beetles and bugs

#### Structure and material properties of photomechanic IR receptors

**Figure 1:**
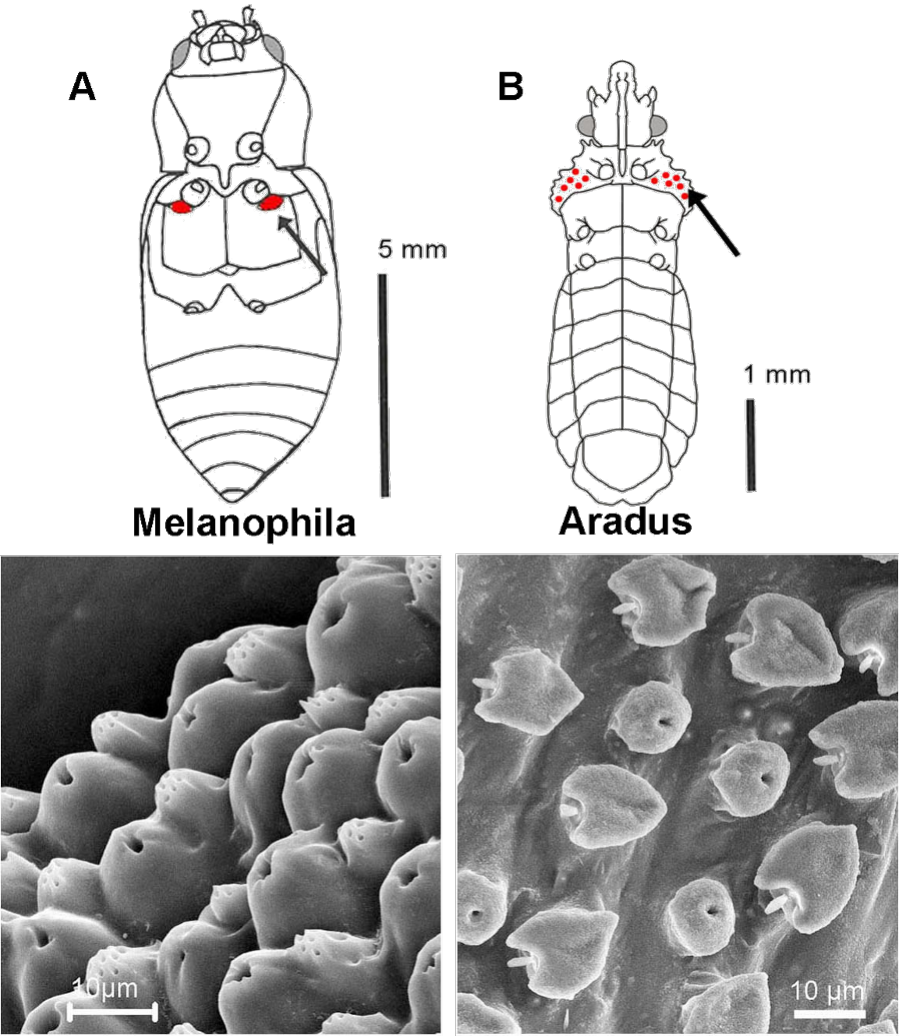
Infrared receptors in *Melanophila* beetles (**A**) and pyrophilous bug species of the genus *Aradus* (**B**). In *Melanophila* about 70 dome-shaped IR receptors which are accompanied by porous wax glands (shown below) are densely packed at the bottom of a thoracic pit (indicated in red and by the arrow). In *Aradus* bugs about a dozen IR receptors (indicated in red and shown below) are interspersed between mechanosensory bristles on the first thoracic segment (arrow) of the bug.

Structure and function of photomechanic insect IR sensillae have been most studied in *Melanophila* beetles. As a special behavioural feature, beetles of both sexes approach forest fires because their brood depends on burnt wood as larval food [5,13–15]. Therefore, it must be postulated that the sensory organs, which are used for fire detection, have been subjected to a strong evolutionary pressure, especially with regard to sensitivity. The individual IR receptors (called sensilla in insects) are situated in two pit organs, which are located on the third thoracic segment. Each IR organ houses about 70 IR sensilla, which are closely packed together at the bottom of the pit [16] (Figure 1A). In pyrophilous bugs of the genus *Aradus*, about a dozen IR sensilla are located on the lateral sides (the so-called propleurae) of the prothorax (Figure 1B). Here, receptors are loosely interspersed between the common hair mechanoreceptors, which cover the entire outer body surface of the bug [12] (Figure 1B).

**Figure 2:**
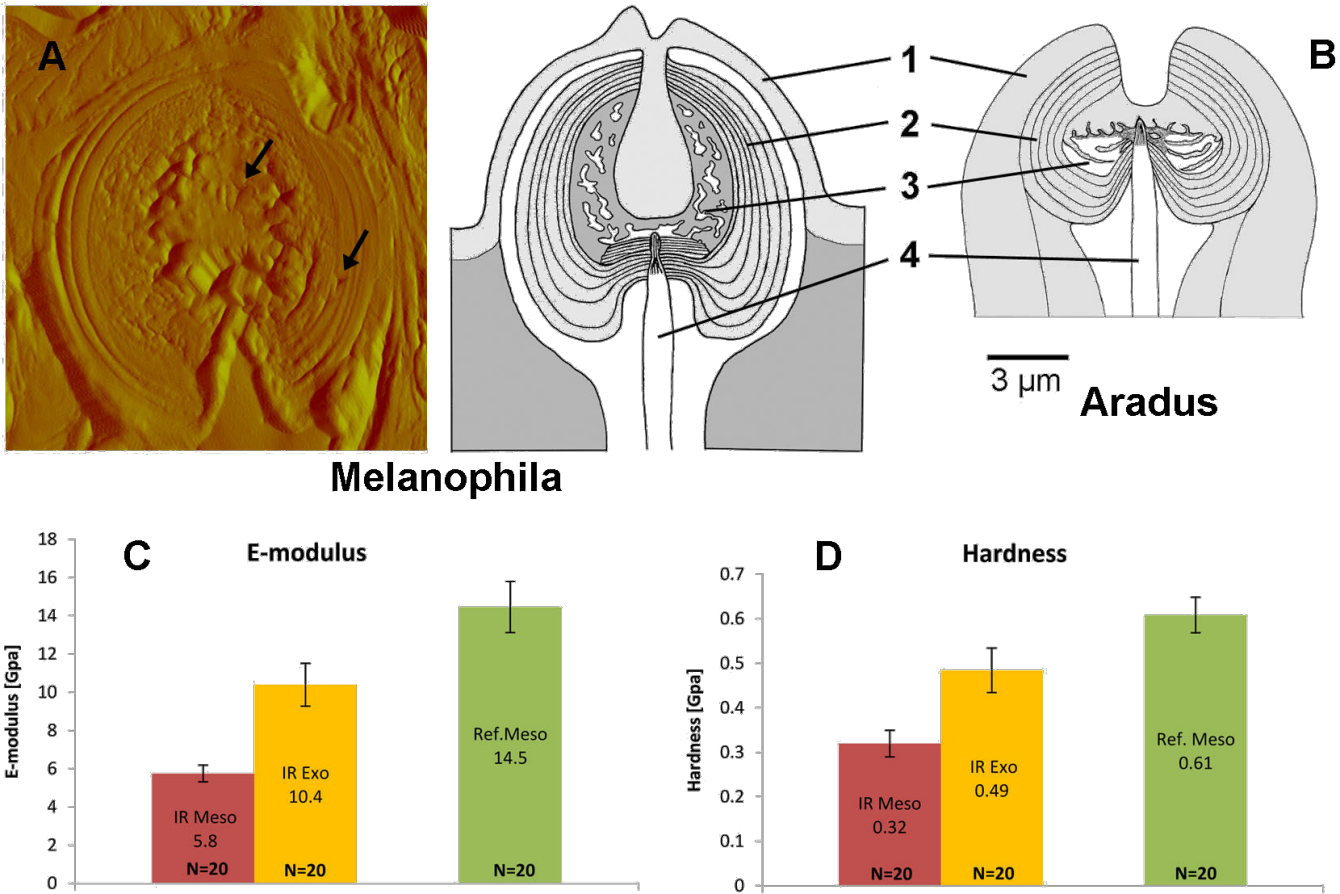
Schematic drawings of single IR receptors of *Melanophila* (A) and *Aradus* (B). 1: outer exocuticle; 2: exocuticular shell of the inner sphere; 3: microfluidic core; 4: tip of mechanosensory dendrite. For the *Melanophila* sensillum a topographical scan made with the tip of the nanoindenter is also shown on the left. Arrows point to indentations in the exocuticular shell of the sphere ("IR Exo" in C and D) and in the mesocuticle of the core ("IR Meso" in C and D). (C) and (D): E-modulus and hardness of different regions of the *Melanophila* sensillum measured by nanoindentation (for details see “*Experimental” section*). Twenty indents were made in each region. "Ref. Meso": reference mesocuticle outside of IR receptor.

From the outside, a single IR sensillum in *Melanophila* beetles and *Aradus* bugs can be recognized by a hemispherical dome with a diameter of about 12–15 µm. The dome consists of a thin cuticle, which represents the outer boundary of a spherical internal cavity. The cavity is almost completely filled with a tiny cuticular sphere with a diameter of about 10 µm (Figure 2A and Figure 2B). Based on transmission electron microscopical observations, it has been observed that the internal sphere consists of at least two different zones: (i) An outer lamellated zone consisting of exocuticle. The lamellated appearance is caused by many layers of chitin fibres with a periodically changing orientation, and (ii) an internal microfluidic core. From below, the sphere is innervated by a single sensory cell. The outermost tip of the sensory dendrite is anchored in the fluidic core. All morphological as well as all physiological data available so far have demonstrated, that this cell is a ciliary mechanoreceptor [1,12,16].

Local testing of different cuticular regions of the *Melanophila* sensillum with a nanoindenter has revealed that the mechanical properties of the exocuticular shell of the sphere and the mesocuticle of the microfluidic core are different: Modulus as well as hardness of the shell are significantly higher. Interestingly, modulus and hardness of reference mesocuticle outside the IR pit organs is much higher than those of the mesocuticle inside the sphere. This points to a specialization of the material inside the sphere (Figure 2C, Figure 2D).

#### Receptor function

Currently, two models of sensillum function can be found in the literature. However, these models are inconsistent with each other. (i) More than 12 years ago, a so-called photomechanic principle was established [17–18]. The authors proposed that the biomolecules (i.e., proteins and chitin) in the cuticular sphere strongly absorb mid-IR radiation. In a way not described in detail, the resulting thermal expansion of the cuticular sphere is measured by the mechanoreceptor. (ii) Assuming the presence of a large air-filled cavity inside the sphere, Evans [19] proposed in a second model that IR radiation enters this cavity by a small apical waveguide with a diameter of about 1.5 µm. Due to the absorption of IR photons at the inner cuticular walls of the cavity, the enclosed air is heated up and expands. In a way not further specified the resulting increase in gas pressure should stimulate the mechanoreceptor.

Our recent findings have clearly demonstrated that the thoracic infrared (IR) sensilla of the pyrophilous jewel beetle *Melanophila acuminata* most likely have evolved from hair mechanoreceptors (sensilla trichodea) [1]. Hair mechanoreceptors, which can be found in any insect, have bristles of different length. In insects the pressure-sensitive dendrite of a single mechanosensitive sensory cell innervates the base of the bristle. As a result, even a slight bending of the bristle stimulates the tip of the dendrite [20]. Compared to a hair mechanoreceptor, a photomechanic IR sensillum in *Melanophila* beetles and *Aradus* bugs shows the following special features (Figure 2): (i) The formation of a complex cuticular sphere instead of a bristle; the sphere consists of an outer hard exocuticular shell as well as an inner relatively soft cavernous mesocuticular part forming a microfluidic core inside the sphere. (ii) The enclosure of the dendritic tip of the mechanosensitive neuron inside the inner core in a liquid-filled chamber. Most probably, IR radiation absorbed by the proteins, the chitin fibres, and the water of the sensillum heats up the sphere, which immediately causes thermal expansion especially of the liquid inside the microfluidic core. Because the shell of the sphere consists of stiff exocuticle additionally reinforced by many layers of chitin fibres, the only compliant structure in the sphere is the membrane of the tip of the mechanosensitive dendrite. Due to the air-filled tracheal system inside the body of the beetle, most of the cell body of the mechanosensitive cell is always at ambient pressure. Only the outermost tip of the sensory dendrite of the mechanosensor (Figure 2) is enclosed inside the sphere. Therefore, any increase in pressure inside the sphere will cause a cross compression of the dendritic tip which is compensated by the cell volume below the sphere. By deflection of the dendritic membrane in the range of a few nanometers, stretch-activated ion channels will be opened. A slow pressure compensation between the inner core of the sphere and the outer environment most probably is achieved by fine nanocanals which run through the outer shell of the sphere. Thus, like the integrated leak in a Golay detector (see below), relatively slow changes of the ambient temperature can be compensated. Hence, we propose that an IR sensillum acts as a microfluidic converter of infrared radiation which leads to an increase in internal pressure inside the sphere, which is measured by the mechanosensitive neuron. The principle of transforming IR radiation into a bioelectrical signal has been termed photomechanic. Most probably photomechanic IR sensilla have been developed independently in beetles and bugs. This provides strong evidence that mechanoreceptors are promising candidates for the development of IR sensors. Because the products of the evolutionary process are very similar in *Melanophila* beetles and *Aradus* bugs [12], it can be proposed that a photomechanic IR sensillum represents an optimized biological sensory system for mid IR radiation.

### Model for the IR receptor

#### Golay sensor

**Figure 3:**
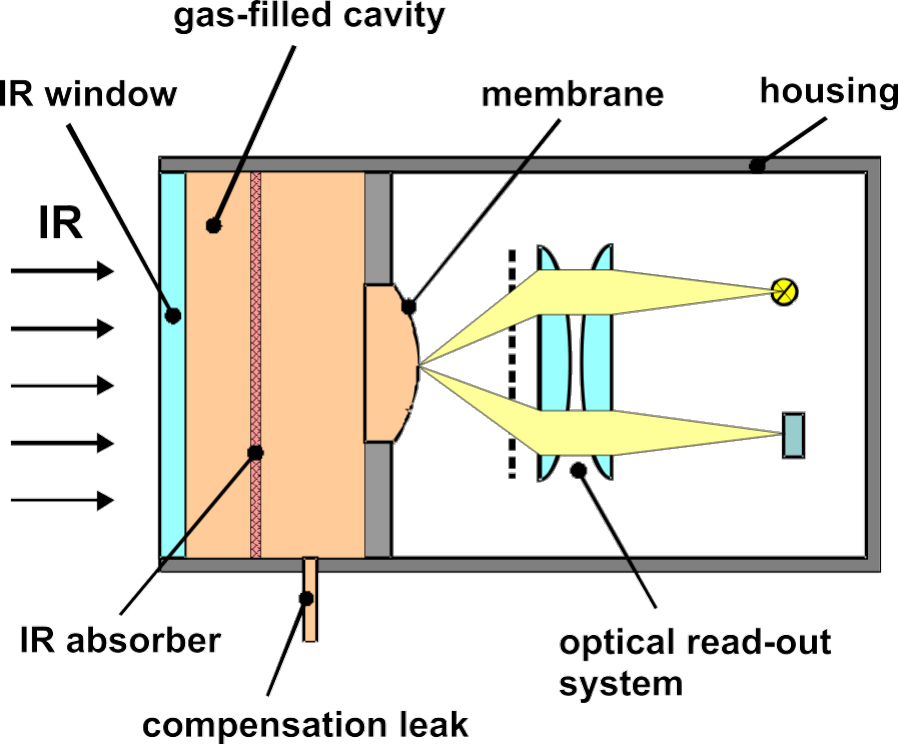
Principle of a gas-filled Golay sensor with optical read-out. For explanations see text.

After the clarification of the function of the biological IR sensor of the beetle we now analyze its principle mathematically to be able to design a technical sensor. For this purpose we investigated the pneumatic Golay sensor [21] as a simple model, see Figure 3. This sensor consists of an internal gas-filled cavity, which is closed on one side by a window and on the other side by a thin membrane. IR radiation enters through the window and heats up the gas by absorption. The deflection of the membrane caused by the expanding gas can be read by an optical system [21], a capacitive detector [22], or a tunneling displacement transducer [23]. To enhance the IR absorption in the gas, the cavity is equipped with an additional absorber. Reflecting walls of the cavity are another means to enhance the absorption. The temperature changes of the gas caused by the absorbed IR radiation are in the range of mK. Because of slow variations of the ambient temperature in the range of a few K, it is absolutely necessary to integrate a leak, which compensates this influence due to an exchange of the gas with a reference volume.

#### Sensor model of the IR receptor

**Figure 4:**
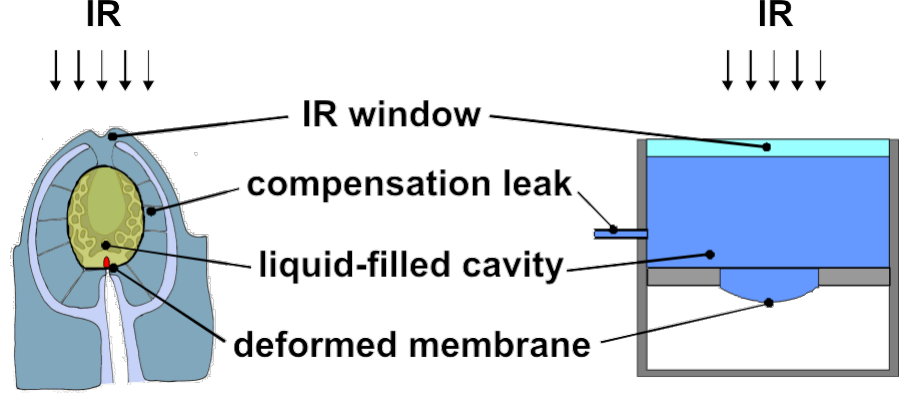
Comparison of the sensillum (left) with the model of the sensor (right).

The model of the sensor, which we want to analyze, is shown in Figure 4. Similar to the sensillum, the sensor contains an internal water-filled cavity. A major difference between the biological sensillum and the Golay sensor is the internal medium, a gas in the traditional Golay sensor, and a fluid in the sensillum. The cavity of the technical sensor is etched into a silicon wafer and is closed on the one side by a window (Suprasil, [24]) and on the other side by a thin silicon membrane. The IR radiation being absorbed produces a change in pressure or volume due to the change of the state of the water. The deflection of the membrane caused by this pressure increase can be read out by, e.g., a capacitive detector or a tunneling displacement transducer.

### Pressure increase of the cavity and deflection of the membrane

For calculating the change of the liquid pressure in the cavity based on the temperature profile, the equation of state must be solved. Because the pressure in the cavity depends upon two independent variables, temperature and volume, its total change is given by

[1]
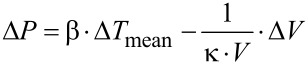


with Δ*P*: Pressure increase, β = (∂*P*/∂*T*)_V_: isochoric tension coefficient, Δ*T*_mean_: mean temperature increase averaged over the cavity volume, κ = −(1/V)·(∂V/∂P)_T_: isothermal compression coefficient, *V*: volume of the cavity, Δ*V*: volume increase. For water (25 °C, 1 bar), β = 5.68·10^5^ Pa/K and κ = 4.5·10^−10^ Pa^−1^ [25].

The increase of the volume Δ*V* of the cavity results in a tiny deflection of the membrane. The deflection *y* of this membrane caused by a pressure difference can be calculated as a function of the radial distance *r* with the shell theory [26]

[2]
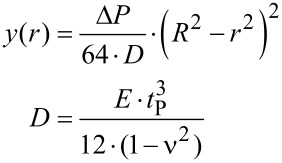


with *R*: radius of the membrane, *D*: flexural stiffness of the membrane, *E*: Young’s modulus, *t*_P_: thickness of the membrane, ν: Poisson’s ratio.

Equation 2 is a good approximation for small membrane deflections, i.e., *y*_max_/*t*_p_ < 1. The volume Δ*V* can be calculated in relation to the pressure increase Δ*P* by using Equation 2

[3]
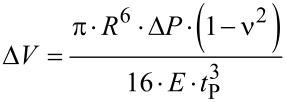


The combination of Equation 2 and Equation 3 yields a relationship for Δ*P*, which considers the influence of temperature and volume.

[4]
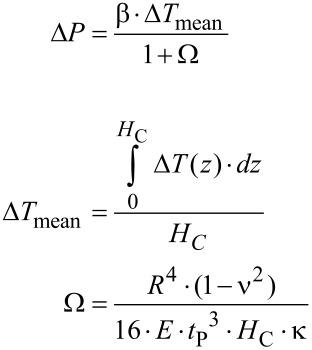


with *H*_C_: height of the cylindrical cavity, Δ*T*(*z*): temperature as a function of the axial coordinate *z* of the cavity and time, Δ*T*_mean_: mean temperature increase averaged over the cavity.

The factor Ω characterizes the change of state of the liquid inside the cavity due to a temperature increase: For Ω → 0, which corresponds to an extremely hard membrane, the change of state is isochoric with a maximal pressure increase. For Ω → ∞, as for an extremely soft membrane, the change of state is isobaric with maximal volume increase. The transition between these two cases is at Ω ≈ 1. The maximal deflection *y*_max_ of the circular membrane can be calculated as a function of the factor Ω by using Equation 2 and Equation 4.

[5]
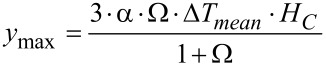


with α = β·κ: isobaric thermal expansion coefficient.

Equation 4 and Equation 5 can be also used for a gas-filled cavity. In this case the isochoric tension coefficient β and the isothermal compression coefficient *κ* can be calculated from the ideal gas law

[6]
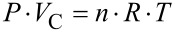


with *n*: number of moles of gas in the cavity, *R*: universal gas constant.

Equation 6 yields the following expression for the isochoric tension coefficient β, the isothermal compression coefficient κ and the isobaric thermal expansion coefficient α

[7]
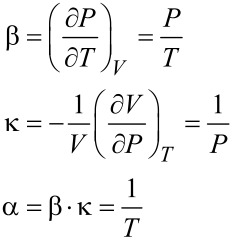


If the change of state is small compared to the initial state *T*_0_ = 300 K and *P*_0_ = 10^5^ Pa, these values result in β ≈ 330 Pa/K, κ ≈ 1.0 · 10^−5^ Pa^−1^ and α ≈ 3.3 · 10^−3^ K^−1^.

The calculation of the mean temperature increase Δ*T*_mean_ in Equation 4 depends strongly on the boundary conditions. When an adiabatic cavity is assumed without any heat loss through the glass window or the cavity wall, the highest possible mean temperature increase Δ*T*_mean_ results.

[8]
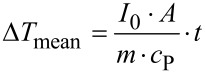


with: *I*_0_: IR power density, *A*: cross-section surface of the cavity, *m*: mass of fluid inside the cavity, *c*_P_: heat capacity of the fluid inside the cavity, *t*: time.

However, in reality the heat loss cannot be neglected. The heat loss through the IR window can be calculated for defined boundary conditions. A high IR absorption coefficient of the liquid in the cavity causes the absorption of the IR energy within a very small absorption zone. For water as a liquid with a very high IR absorption coefficient, a formula for the temperature profile Δ*T*(*z*) could be derived [27] on the assumption of a infinitely thin boundary layer between the glass window and the water. This allows a numerical solution of the mean temperature increase in Equation 4 taking into account the heat loss through the IR window. In the case of water as liquid up to 50% of the absorbed energy may be lost through the window by heat conduction [27]. For liquids with lower absorption coefficients than water, the mean temperature increase must be calculated numerically by finite element methods.

Gases always have a significant lower absorption coefficient which requires the application of an additional absorber such as plastic, aluminium, antimony or lead [21]. When the absorbing film is directly on the inner surface of the window, the assumption of a boundary layer as in the case of water is valid.

### Evaluation of different liquids and gases as fluids inside the cavity

#### Water

**Figure 5:**
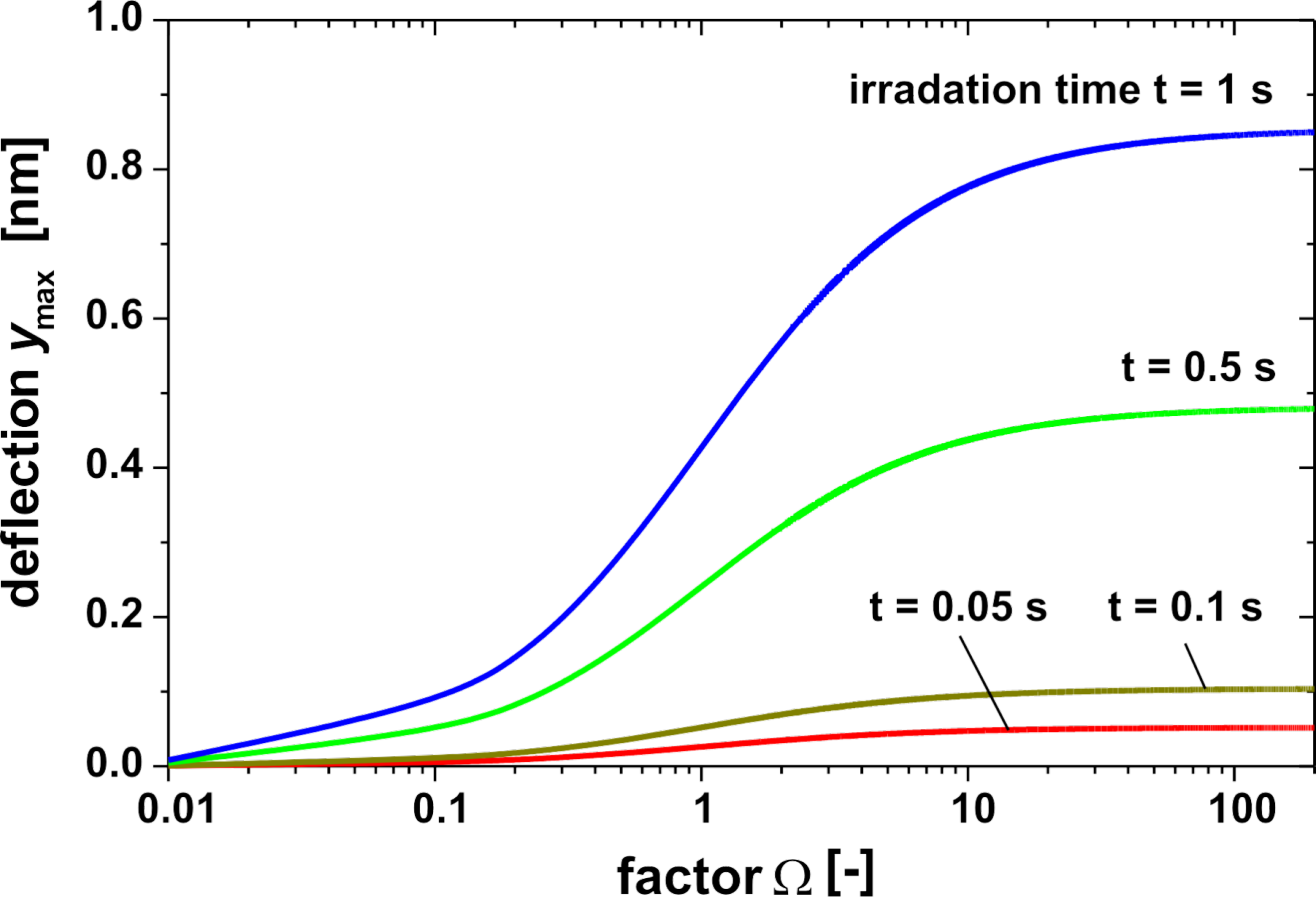
Maximum central deflection *y*_max_ of a circular membrane as function of factor Ω and irradiation time for a water-filled cavity. IR power density = 10 W/m^2^, diameter and height of the cavity = 0.5 mm.

Figure 5 shows the maximal deflection as a function of the factor Ω according to Equation 5 for an IR power density of 10 W/m^2^ (IR window without absorption loss assumed) for a cavity with a height and a diameter of 0.5 mm filled with water. These values were taken as a reference. Obviously, the maximal deflection is achieved for the isobaric case with a very soft membrane. Due to the very low deflection of less than 1 nm, a capacitor for the read-out must be extremely sensitive. As an alternative an optical read-out or the use of a tunneling displacement transducer may be useful.

With regard to Equation 4 and Equation 5, the maximal deflection of the membrane can be increased by using a liquid with a high thermal expansion coefficient α and a high mean temperature increase Δ*T*_mean_ due to a low product of heat capacity and density in a small cavity. Additionally, the IR window and the cavity material should have a low heat conduction to avoid heat losses during measurement.

#### Hydrocarbons

The inner pressure chamber of the sensillum is filled with a liquid, which consists mostly of water. Regarding the thermal expansion coefficient, water is not an optimal liquid. Hydrocarbons such as *n*-pentane and toluene, as well as methanol, which are also used as fluids in thermometers, are more appropriate. Table 1 shows the material properties of different hydrocarbons and carbon dioxide in comparison to water.

The thermal expansion coefficients of the hydrocarbons in Table 1 are about eight times higher compared to water so that the deflection of the membrane increases by this factor. The heat capacity and density of hydrocarbons is about 40–50% lower in comparison to water, which results in a higher mean temperature increase Δ*T*_mean_ for the same amount of stored energy.

**Table 1:** Comparison of material properties of different hydrocarbons, water and CO_2_ (300 K, 10^5^ Pa). The absorption coefficients α_OPT_ were calculated in the bandwidth 3–5 µm using data from [28–30].

	α [10^−5^ K^−1^]	κ [10^−10^ Pa^−1^]	ρ [kg/m^3^]	λ [W/(m·K)]^a^	c_p_ [kJ/(kg·K)]	α_OPT_ [cm^−1^]

water	26	4.5	998	0.61	4.18	1140
*n*-pentane	158	25.0	627	0.11	2.31	73
toluene	107	9.1	870	0.14	1.72	94
CO_2_ (gas)	330	10^5^	1.76	1.64·10^−2^	0.85	—^b^

^a^heat conductivity; ^b^For CO_2_ gas an infinitely thin absorption zone is assumed.

The absorption coefficients in Table 1 were calculated in the atmospheric mid-wave window between 3–5 µm using data from [28–30]. Compared to water the absorption coefficients of hydrocarbons are up to two orders of magnitude lower. For this reason, the temperature distribution along the cavity axis is more uniform compared to water.

**Figure 6:**
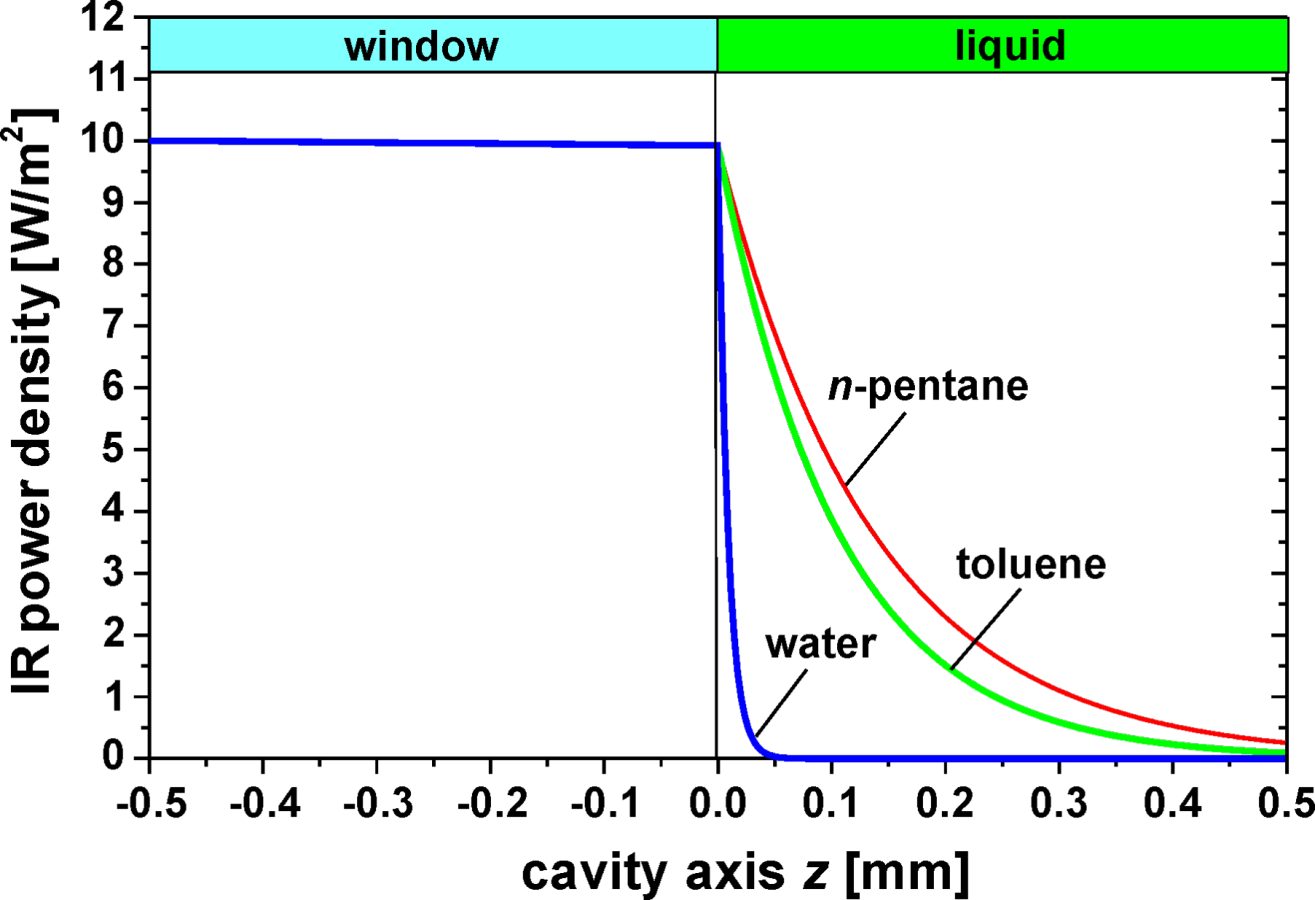
IR power density as function of the cavity axis for different liquids. Suprasil^®^ 300 was used as the material for the window with an absorption coefficient of 0.16 cm^−1^ [23]. For gas, a thin absorbing film on the inner glass surface is assumed where all the radiation is absorbed.

**Figure 7:**
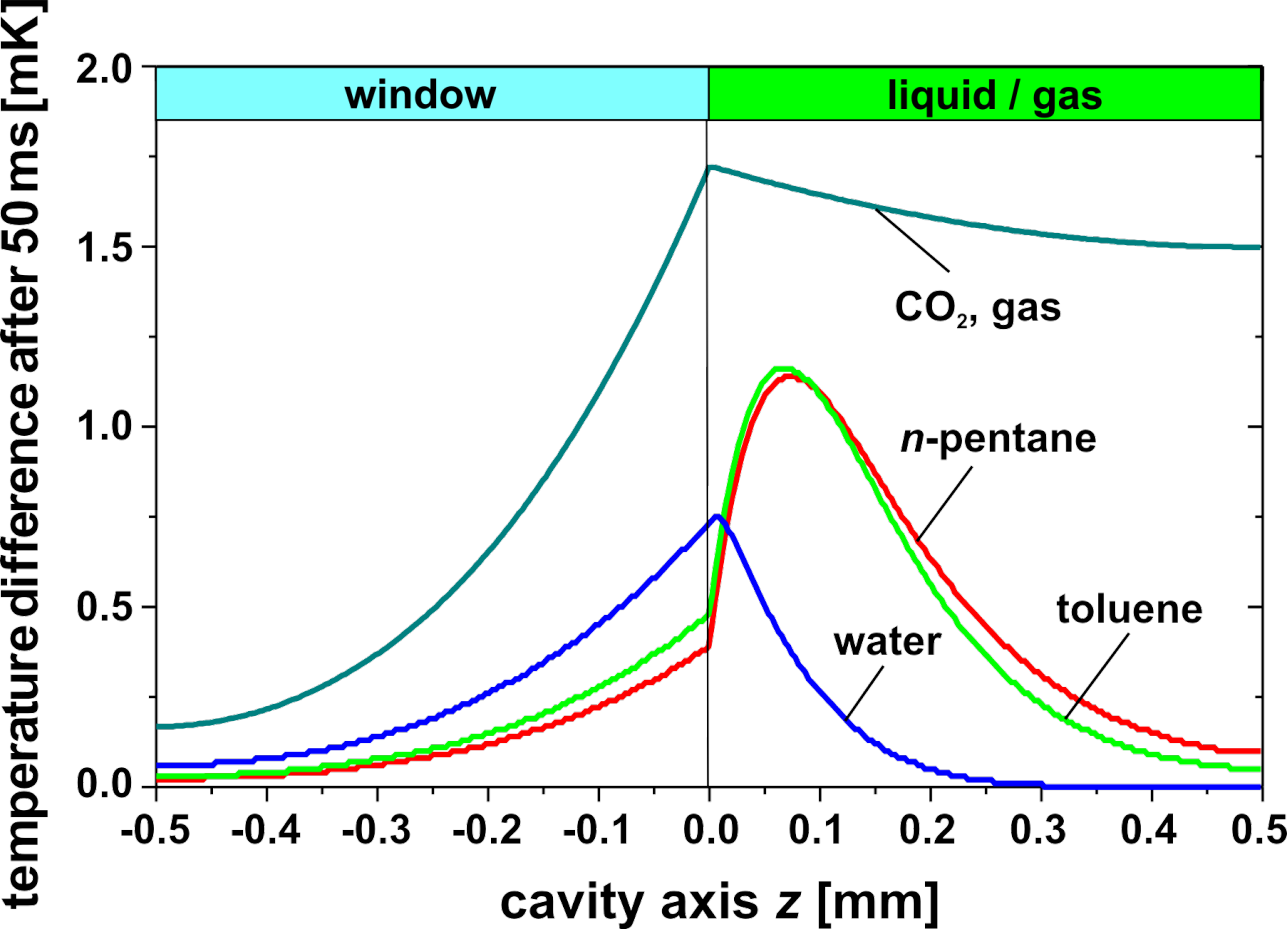
Temperature distribution along the cavity axis 50 ms after the onset of irradiation for different liquids with an IR power density at the outer window surface of 10 W/m^2^, see Figure 6.

For the evaluation and comparison of the different liquids regarding the maximum membrane deflection in Equation 5, the mean temperature increase Δ*T*_mean_ has to be calculated. An IR power density of 10 W/m^2^ at the outer window surface in a bandwidth of 3–5 µm and an irradiation time of 50 ms is used as a reference. For simplification it is assumed that the IR power density is independent from the wavelength in this bandwidth. Figure 6 shows the decrease of the power density as a function of the cavity axis coordinate *z* for different liquids. Obviously the decrease of the power density in water occurs only within a thin absorption zone directly behind the window, whereas in the case of hydrocarbons the entire depth of the cavity contributes to absorption. The power density profiles in Figure 6 were used to numerically calculate the temperature distribution after 50 ms in Figure 7. Due to the thin absorption zone in water, the maximum temperature appears directly behind the window resulting in heat conducting losses through the window. For hydrocarbons, however, the maximum temperature is shifted deeper into the cavity causing smaller heat losses through the window. Based on the temperature profiles in Figure 7, the mean temperature increase Δ*T*_mean_ is calculated in Table 2. Here the advantage of using hydrocarbons instead of water is also obvious.

**Table 2:** Comparison of the mean temperature increase Δ*T*_mean_ , the factor Ω, and the maximum deflection *y*_max_ of the membrane in different liquids and CO_2_ gas after 50 ms irradiation of 10 W/m^2^.

	Δ*T*_mean_ [mK]	Ω [-]	*y*_max_ [nm]

water	0.14	6245	0.04
*n*-pentane	0.53	1126	1.26
toluene	0.64	3093	1.03
CO_2_ (gas)	1.57	0.281	2.18

The silicon membrane (*E* = 166 GPa, ν = 0.28 [31]) has a diameter of 0.5 mm and a thickness of 1 µm, resulting in a factor Ω of 1000 to 6000 for the different liquids in Table 2. Regarding Figure 4 this is the isobaric case, and due to the high factor Ω a softer membrane will not result in a larger deflection of the membrane. With the data of the membrane, the deflection can be calculated by using Equation 5, see Table 2. A value of only about 1 nm results for the hydrocarbons. The increase of the deflection in case of the hydrocarbons by a factor 10 compared to water as liquid can be explained by an increase of the mean temperature of about factor 4–5 and an increase of the expansion coefficient by the factor 5–7. It can be assumed as a first approximation, that the deflection is proportional to the IR power density and the irradiation time.

If a pure liquid is used the thermal and optical properties are coupled, that means a liquid with an optimal product of density and heat capacity may have a low IR absorption, which makes this fluid disadvantageous for the application. A decoupling of thermal and optical properties can be achieved with a matrix of a good absorber, e.g., a meshwork of plastic or tiny plastic beads, or a dye immersed in the fluid. The plastic matrix or a dye absorbs most of the radiation and transfers the absorbed energy to the fluid, which provides optimal thermal properties and a large thermal expansion. The choice of the plastic matrix must ensure a large surface-to-volume ratio and a low product of density and heat capacity. The decoupling of the thermal and optical properties permits the determination of an optimal absorption coefficient approximately for each cavity depth to absorb the IR energy completely in the cavity without any unused fluid volume.

#### Gas

**Figure 8:**
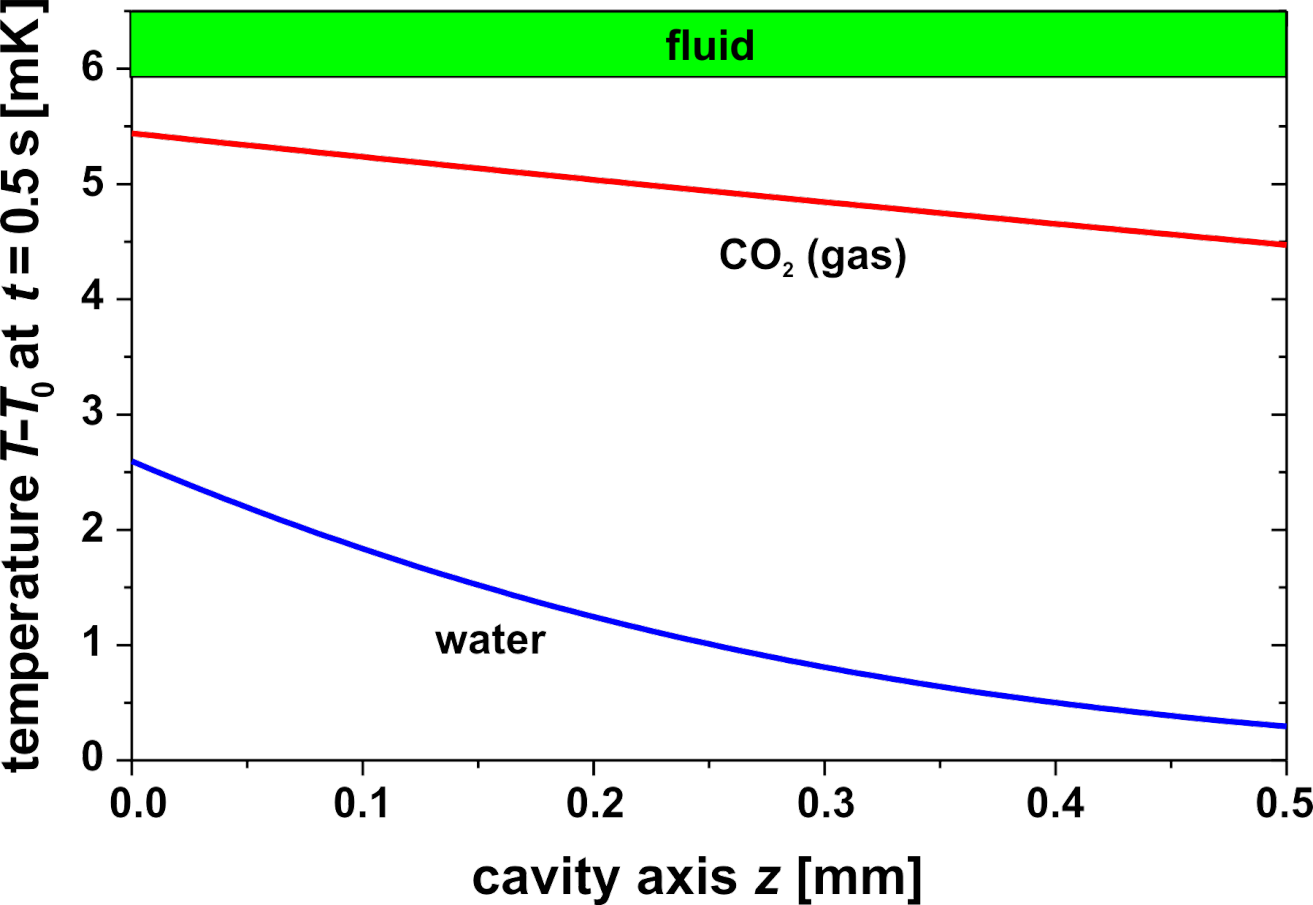
Temperature distribution along the cavity axis *z* 0.5 s after the onset of irradiation for a water-filled and a CO_2_-filled cavity with an IR power density at the outer window surface of 10 W/m^2^. In the case of gas, a thin IR absorber film is assumed on the inner glass surface where all the IR energy is absorbed.

**Figure 9:**
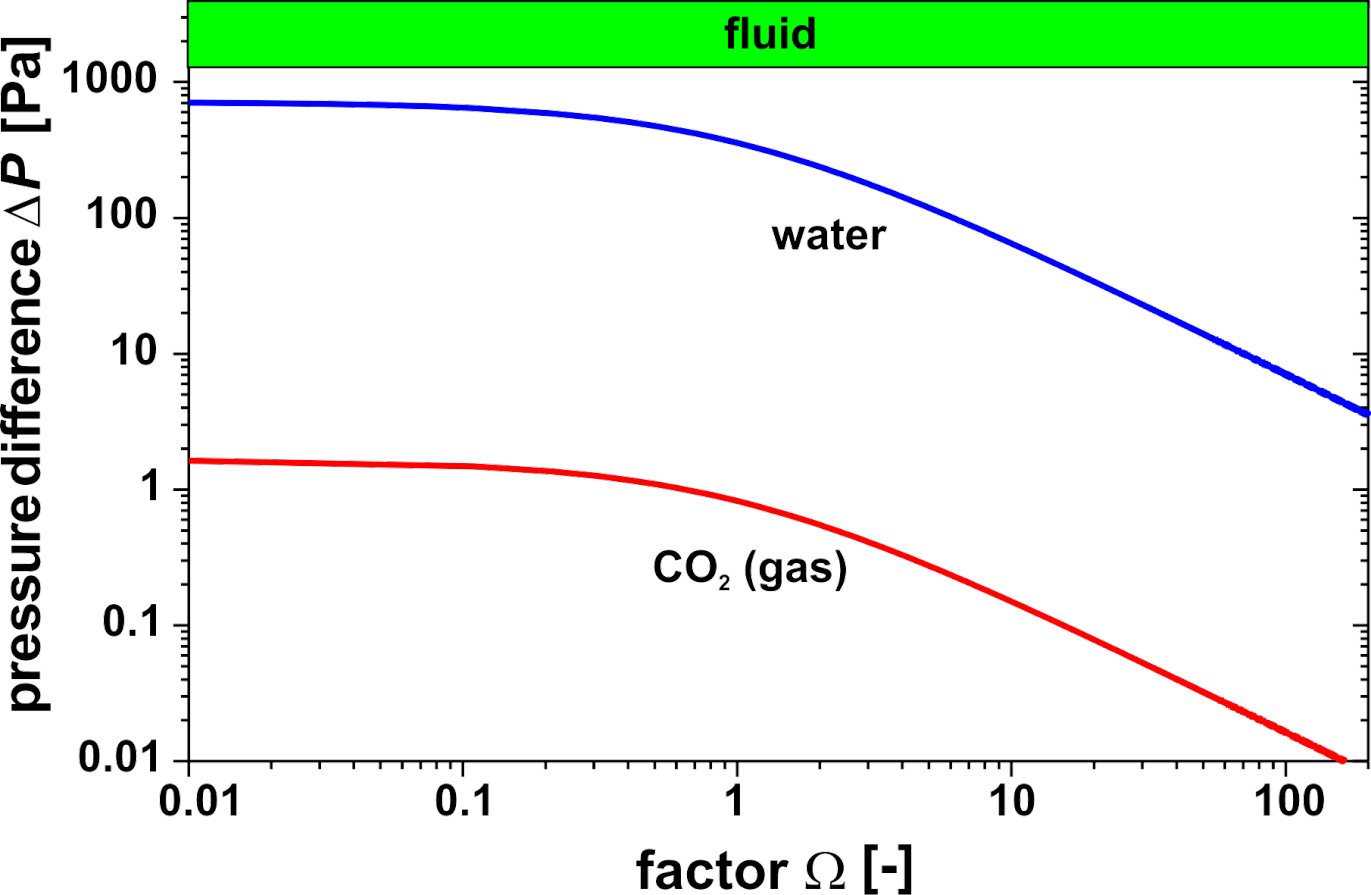
Pressure difference Δ*P* of a circular membrane at *t* = 0.5 s as a function of factor Ω for a water-filled and CO_2_-filled cavity. IR power density 10 W/m^2^, diameter and height of the cavity: 0.5 mm.

**Figure 10:**
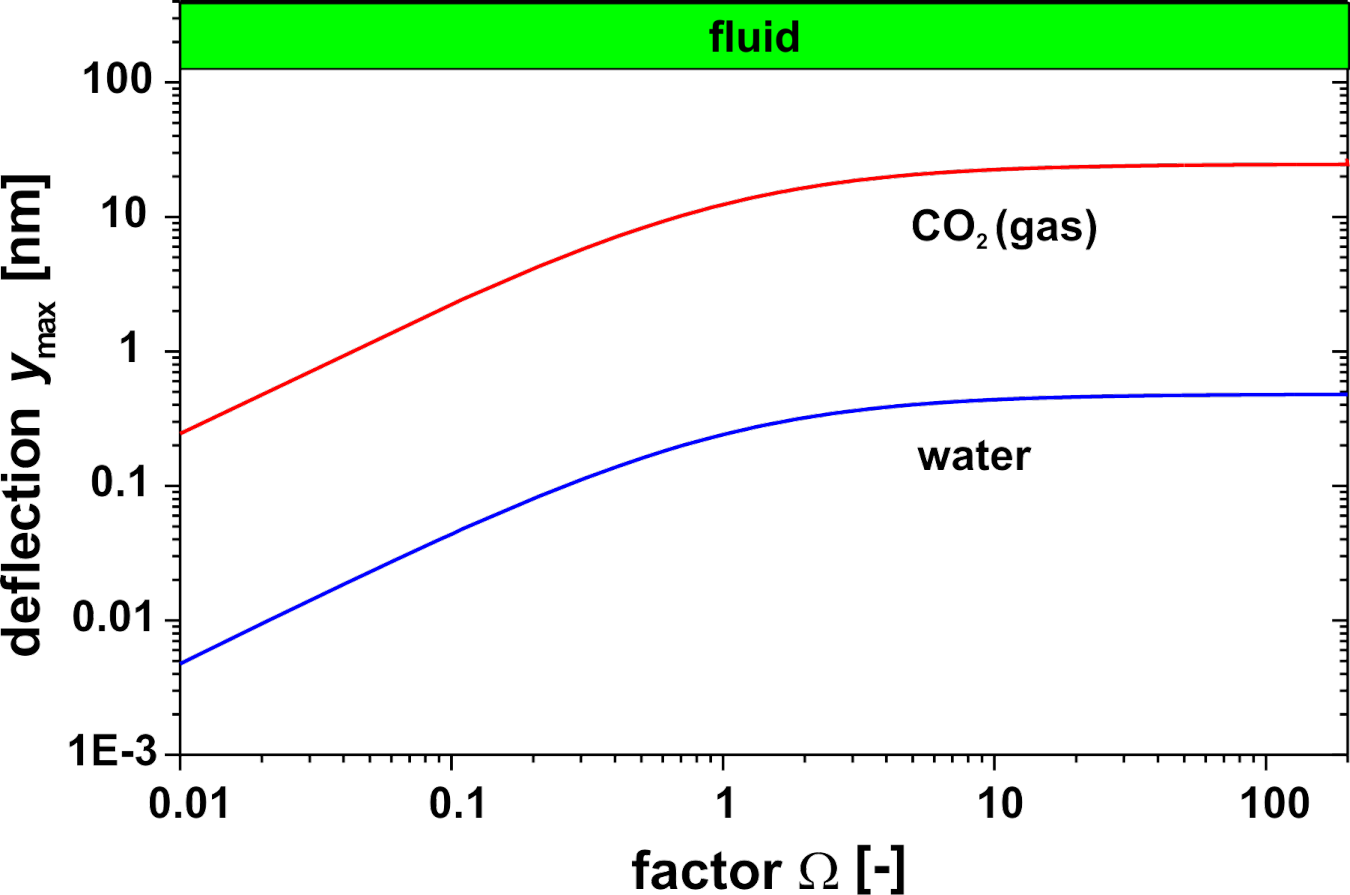
Maximum central deflection *y*_max_ of a circular membrane at *t* = 0.5 s as function of factor Ω for a water-filled and CO_2_-filled cavity. IR power density 10 W/m^2^, diameter and height of the cavity: 0.5 mm.

Using gas instead of water in the cavity as in the well known pneumatic Golay sensors yields different results due to the different material properties, in particular density ρ, heat capacity *c*_p_ and the coefficients β, κ, α in Equation 7. For a better comparison with the water-filled cavity, it is also assumed that a thin zone due to a thin absorbing film on the inner glass surface exists where all the radiation is absorbed and this energy heats up the gas by heat conduction. The resulting temperature profile for water and gas 0.5 s after the onset of IR irradiation is shown in Figure 8 based on the same IR power density 10 W/m^2^. Mainly due to the lower product of density and heat capacity, the temperature increase in the gas is higher; the mean temperature increase Δ*T*_mean_ is in the case of water 1.2 mK and 4.8 mK in the case of gas. Using Equation 4, this causes, e.g., for Ω = 1 a pressure difference Δ*P* of 350 Pa in the case of water but only 0.8 Pa in the case of gas (Figure 9). However, the maximal deflection of the membrane in the case of gas is 12 nm compared to 0.2 nm in the case of water, see Figure 10. This surprising fact can be explained by the higher mean temperature increase and especially by the higher thermal expansion coefficient in the case of gas compared to water. The temperature distribution 50 ms after the onset of IR irradiation is shown in Figure 10 comparing gas versus water and hydrocarbons. In this case the maximal deflection of the membrane is about 2 nm compared to about 1 nm in the case of hydrocarbons (Table 2).

Using different gases will not change the result noticeably, because only the individual gas constant and the heat capacity are variable. However, using a gas with a low heat conductivity such as argon or xenon will reduce the radial heat losses in the cavity wall and yield a higher temperature increase.

### Read-out of the membrane deflection

The calculated deformation of the membrane in the case of water as liquid and the deformation of the tip of the dendrite in the sensillum have the same magnitude. In mechanoreceptors such as the sensillum, deformations of the tip of the dendrite of only 0.1 nm, corresponding to an energy of 10^−19^ J, yield a receptor potential [32]. As a consequence of the tiny deformation of the membrane the technical sensor needs a read-out system that is able to detect deformations in the nm-range, such as interferometry, tunneling contacts [23], or a capacitive position sensor with nanometer resolution [33].

### Compensation leak

**Figure 11:**
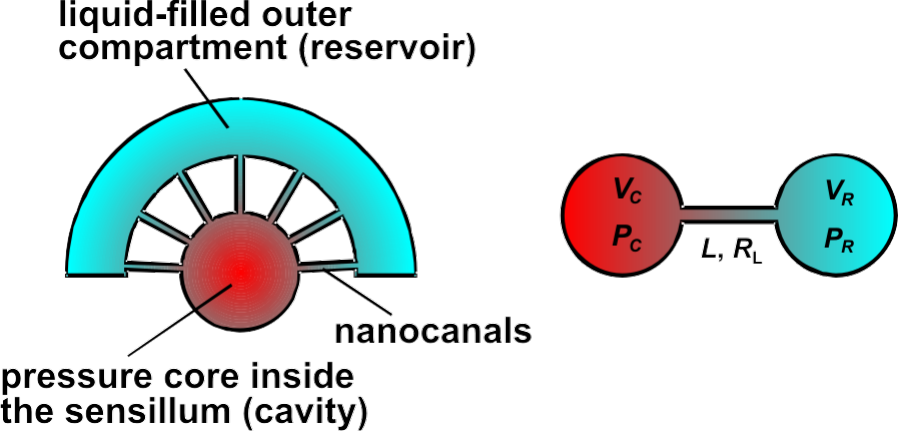
Left: model of the pressure core in the sensillum connected by nanocanals with the outer compartment; right: calculation model for pressure compensation between the core (cavity, volume *V*_C_, pressure *P*_C_) connected by a canal (length *L*, radius *R*_L_) with a reservoir (volume *V*_R_, pressure *P*_R_) and *P*_C_ > *P*_R_.

In the IR receptor of *Melanophila acuminata* the inner sphere is enclosed by a thin layer of liquid in an outer compartment. Therefore the nanocanals in the shell of the sphere allow the exchange of liquid in and out of the microfluidic core in the sphere. Thus, any internal pressure change, which may be caused by the slowly changing ambient temperature, can be compensated. Golay sensors also use such compensation leaks for this purpose [21]. Figure 11 shows a simplified model of the sensillum with the core connected by nanocanals with the outer compartment and the geometric design of such a compensation leak. For the design of a compensation leak a formula will be derived.

For a liquid as fluid a mass balance between the two volumes of the cavity and of the reservoir yields a system of two partial differential equations.

[9]
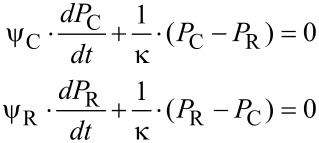


with the following abbreviations:

[10]
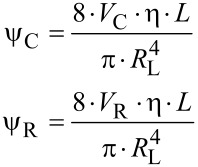


where η: dynamic viscosity, *L*: Length of canal, *R*_L_: Radius of canal.

For the solution of the differential equations in Equation 9, a Hagen–Poiseuille flow in the canal is assumed [34–35].

[11]
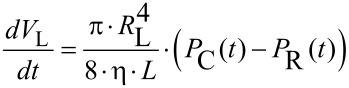


where (*dV*_L_/*dt*): volumetric flow rate in the canal, *P*_C_(*t*), *P*_R_(*t*): time dependent pressures in the cavity and the reservoir.

The solution of the differential equations is given by [35]

[12]
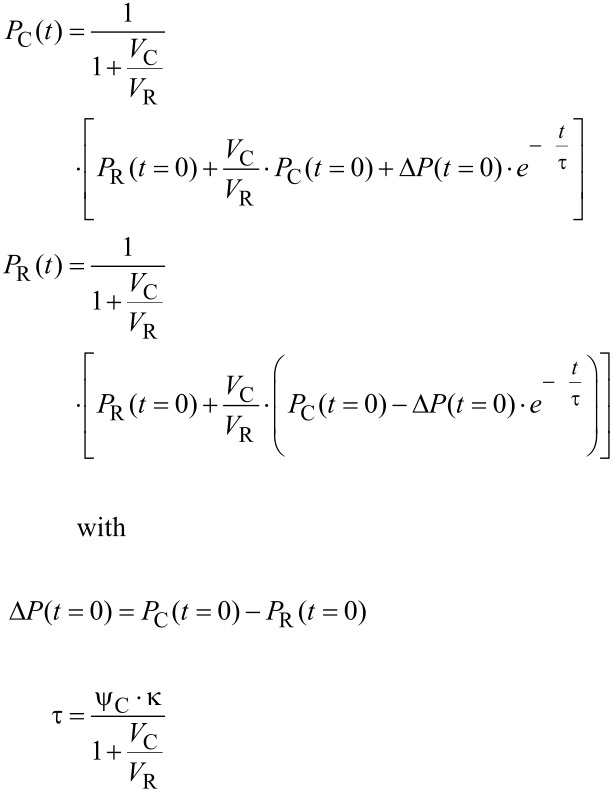


with *t*: time, *P*_C_(*t* = 0), *P*_R_(*t* = 0): initial pressure in the cavity and the reservoir at *t* = 0.

With N similar canals between the cavity and the reservoir, the time constant τ in Equation 12 is reduced by this number

[13]
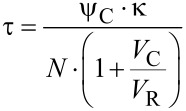


**Figure 12:**
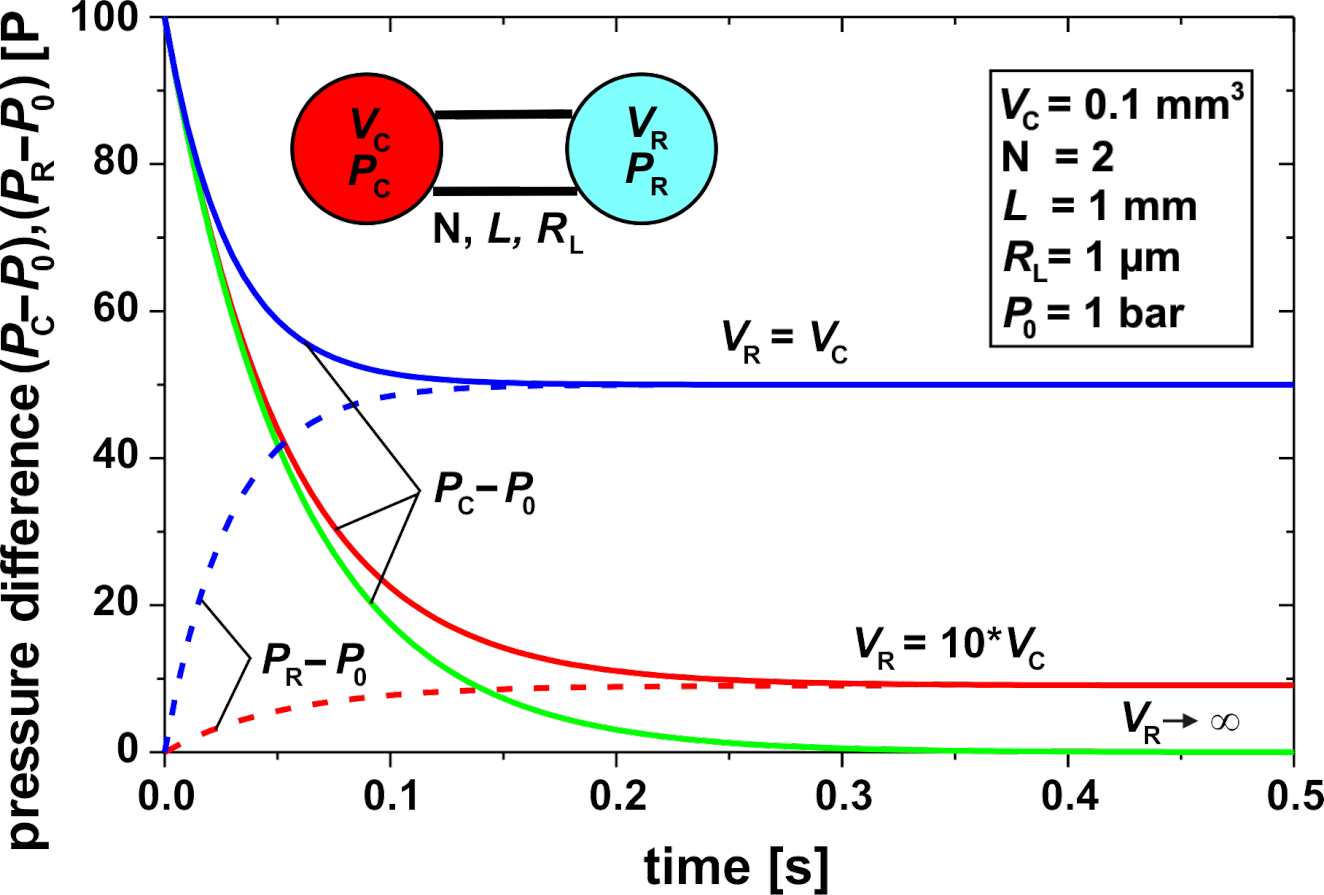
Pressures in the cavity, *P*_C_, and in the reservoir, *P*_R_, as a function of time for a cavity with a volume *V*_C_ = 0.1 mm^3^ connected by two compensation leaks to a reservoir volume *V*_R_ for different values of *V*_R_/*V*_C_, *P*_0_: ambient pressure.

By assuming only little changes of the liquid density, Δρ/ρ 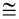 0, Equation 12 and Equation 13 can be used with κ = 1/*P* for a gas. This assumption is justified when *P*_C_(*t* = 0) is similar to *P*_R_(*t* = 0). With *V*_C_ << *V*_R_, i.e., an infinite reservoir, and with gas as the fluid, the time constant τ in Equation 10 is equal to the time constant described by Chevrier et al. in [22] derived with an electrical circuit analogy. Three examples of the pressure release between a cavity *V*_C_ (diameter 0.5 mm, depth 0.5 mm) connected by two compensation leaks to reservoirs *V*_R_ with different size and water as liquid are shown in Figure 12. The initial pressure difference Δ*P* of 100 Pa is, e.g., generated by a Δ*T*_mean_ in the cavity of about 1 mK. The time necessary for pressure compensation is about 0.15 s, when the cavity volume and the reservoir volume are equal, and is increased up to 0.3 s when the reservoir volume is 10 times larger than the cavity volume.

**Figure 13:**
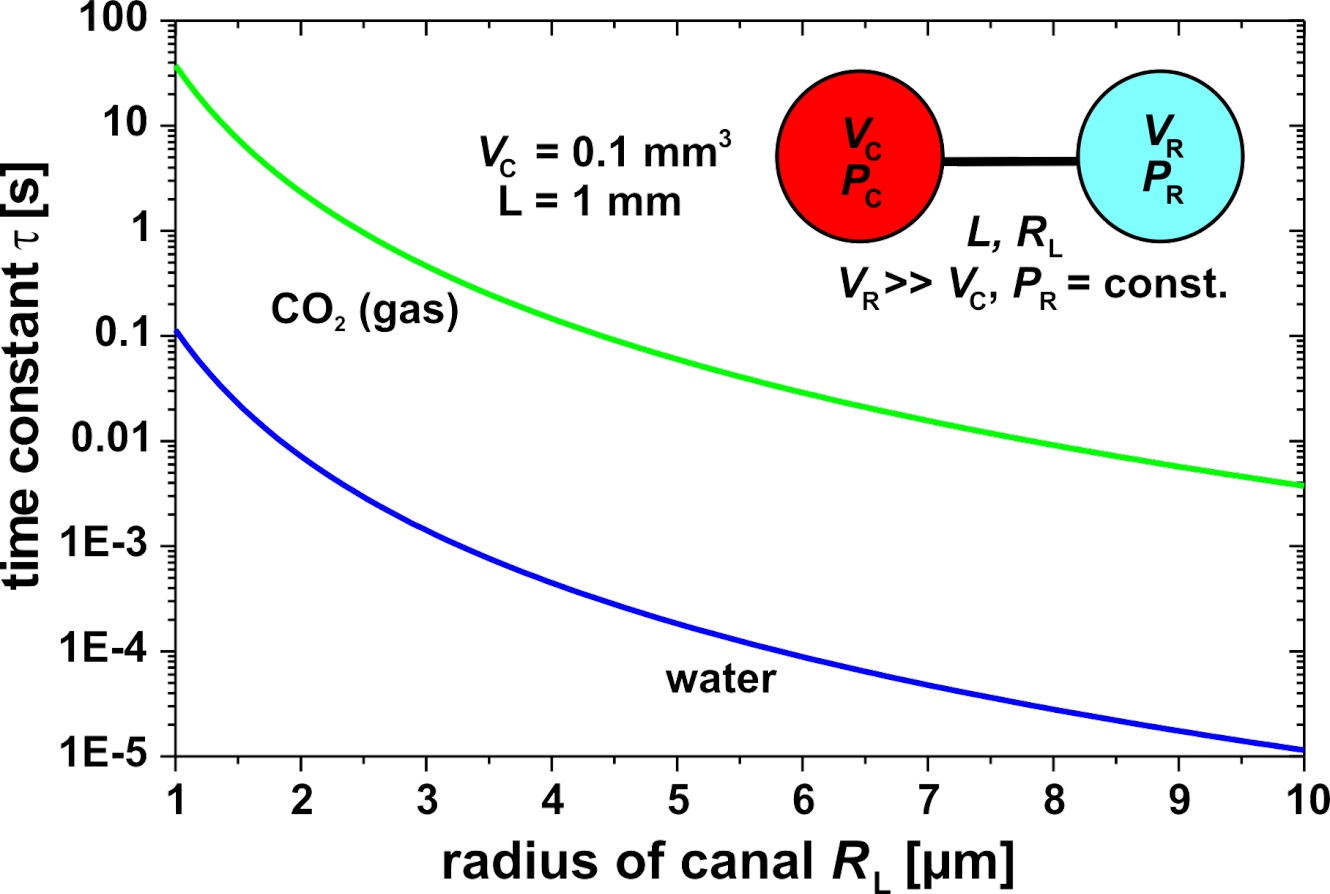
Comparison of time constants for a compensation leak of a cavity filled with water or with CO_2_ gas as a function of the radius of the canal.

The time constant of the compensation leak must be considerably larger than the time constant of the temperature change in the cavity due to an IR signal in order to minimize the effect of the compensation leak on the measurement. It is obvious that the time constant of the compensation leak can be tuned by the geometry and the number of the canals and the ratio of the cavity volume and the reservoir volume. Figure 13 shows the time constants of a compensation leak as function of the radius of the canal and the fluid in the cavity, water or CO_2_ gas. Especially due to the higher compressibility of the gas, the time constant is in this case up to three orders of magnitude higher than in the case of water. When a time constant of 1 s is assumed, then in the case of water the compensation canal needs to have some mm length with a radius smaller than 1 µm. The use of gas instead of water will result in a simpler manufacturing procedure due to the larger radius of the canal.

## Conclusion

Just as in the photomechanic insect IR receptors, our technical model features very small displacements of the membrane in the sensing device in the range of 1 nm or less. The insects, however, have the advantage of possessing a very sensitive mechanoreceptor, the mechanosensitive neuron, which can detect minute deflections of its membrane. It seems that this sensitivity of the beetle is difficult to achieve for a technical sensor.

For our miniaturized technical sensor, an appropriate technical read-out mechanism with a high resolution of 1 nm may be necessary. Currently, a sensor with a capacitor as read-out is near completion in the *center of advanced european studies and research* (caesar), Bonn. As an alternative read-out for displacements in the nm-range, tunneling displacement transducers are under investigation. Several read-out methods will be evaluated, but it has to be stressed that this search must yield a rugged and cost-effective design in order to be able to compete with existing IR sensors.

An obvious method to enhance the displacement of the membrane is to choose liquids with optimal thermal properties, e.g., high thermal expansion coefficient and low heat capacity. Here several liquids permit an improvement. Methanol is a good candidate compared to *n*-pentane and toluene because of easier handling during the filling process of the cavity. The comparison of a liquid-filled cavity with a gas-filled cavity suggests that gas causes a larger deflection of the membrane. However, the final weighing of potential advantages or disadvantages of a miniaturized photomechanic IR sensor filled with a fluid needs further investigation.

Additionally, care has to be taken in selecting appropriate wall materials with the same low thermal conduction necessary to preserve the thermal energy inside the cell. Currently silicon is used due to its well known manufacturing technology for microsystems. As an alternative micro injection molding of plastic materials with low heat conductivity is under investigation.

## Experimental

Morphological methods used are all based on well established light and electron microscopical procedures.

Mechanical tests were conducted in a nanomechanical test system capable of normal loading as well as in situ scanning probe microscopy (SPM) (TriboScope; Hysitron, Minneapolis, USA). Indentation tests were performed by using a three-sided Berkovich diamond tip with a total included angle of 142.3°. A proper area function was established by indenting in a poly(methyl methacrylate) (PMMA) test specimen with known hardness and modulus. Contact depths range from 250 to 1100 nm. The maximum load during indentation was 1,000 µN with loading and unloading rates of 100 µN/s, and a 10 s hold time at peak load to compensate for material creeping and to make sure that most of the plastic deformation was completed. It was repeatedly checked to ensure that longer holding times did not result in differing values. Hardness (*H*) and reduced Young´s modulus (*E*_r_) both were calculated from the unloading portions of the load–displacement curves following well established procedures.
